# Risky sexual practices among youth attending a sexually transmitted infection clinic in Dar es Salaam, Tanzania

**DOI:** 10.1186/1471-2334-8-159

**Published:** 2008-11-19

**Authors:** W Urassa, C Moshiro, G Chalamilla, F Mhalu, E Sandstrom

**Affiliations:** 1 Department of Microbiology and Immunology, Muhimbili University of Health and Allied Sciences, Tanzania; 2 Epidemiology and Biostatistics, Muhimbili University of Health and Allied Sciences, Tanzania; 3 Ilala Municipal Council, Dar es Salaam, Tanzania; 4 Karolinska Institute Stockholm, Sweden

## Abstract

**Background:**

Youth have been reported to be at a higher risk of acquiring STIs with significant adverse health and social consequences. Knowledge on the prevailing risky practices is an essential tool to guide preventive strategies.

**Methods:**

Youth aged between 18 and 25 years attending an STI clinic were recruited. Social, sexual and demographic characteristics were elicited using a structured standard questionnaire. Blood samples were tested for syphilis and HIV infections. Urethral, high vaginal and cervical swabs were screened for common STI agents.

**Results:**

A total of 304 youth were studied with mean age of 21.5 and 20.3 years for males and females respectively. 63.5% of youth were seeking STI care. The mean age of coitache was 16.4 and 16.2 years for males and females respectively. The first sexual partner was significantly older in females compared to male youth (23.0 vs 16.8 years) (p < 0.01). 93.2% of male youth reported more than one sexual lifetime partner compared to 63.0% of the females. Only 50% of males compared to 43% of females had ever used a condom and fewer than 8.3% of female youth used other contraceptive methods. 27.1% of pregnancies were unplanned and 60% of abortions were induced. 42.0% of female youth had received gifts/money for sexual favours. The HIV prevalence was 15.3% and 7.5% for females and males respectively. The prevalence of other STIs was relatively low. Among male youth, use of alcohol or illicit drugs was associated with increased risk of HIV infection. However, the age of sexual initiation, number of sexual partners or the age of the first sexual partner were not associated with increased risk of being HIV infected.

**Conclusion:**

Most female youth seen at the STI clinic had their first sexual intercourse with older males. Youth were engaging in high risk unprotected sexual practices which were predisposing them to STIs and unplanned pregnancies. There is a great need to establish more youth-friendly reproductive health clinics, encourage consistent and correct use of condoms, delay in sexual debut and avoid older sexual partners in females.

## Background

Adolescent, the period in particular between 10 and 25 years involves sexual experimentation that may lead to acquisition of sexually transmitted infections (STIs) and unplanned pregnancies. The risky sexual practices in this age group may include early sexual debut, having multiple sexual partners, engaging in unprotected sexual intercourse, engaging in sex with older partners and consumption of alcohol and illicit drugs. [1-6]

Several studies done in sub Saharan Africa, including Tanzania, have shown a high prevalence of STIs including HIV among youth, with females having higher prevalence compared to males. [3,6-11] Reasons for higher susceptibility of females have been found to be multifactorial and include biological, economical and social demographic factors; mixing patterns among sexual partners, the age difference between male and female sexual partners, with males seeking sexual gratification from younger females and peer pressure. [12-15] In contrast, most studies have indicated that male youth have a higher number of sexual partners than females. [12-15]

We describe the sexual practices in relation to HIV infection among adolescents and youth below 25 years of age attending a youth STI clinic in Dar es Salaam, Tanzania in order to try to understand factors that may facilitate STI acquisition and suggest preventive strategies.

## Methods

The study population was recruited by inviting every third youth aged between 18 and 25 years attending the STI clinic, at the Infectious Diseases Centre (IDC) at the centre of Dar es Salaam city on any particular day between April 2002 and June 2004 to participate. The youth were informed about the objectives of the study and those who gave a written informed consent were recruited. A structured questionnaire was used to obtain social, sexual and demographic characteristics. A comprehensive clinical examination was also done. After individual pre-test counselling, a blood sample was drawn for serological testing for syphilis and HIV infections. History and physical examination including taking of urethral, high vaginal, endocervical swabs or first voided urine samples for detection of possible aetiological agents of selected reproductive tract infections (RTI) using standard microbiological methods. Patients were encouraged to refer their sexual partners for treatment and/or counselling to the same clinic or to any of the other STI clinics in the city.

HIV testing was done using two sequential ELISAs (Enzygnost anti HIV 1+2 Behring Marburg, Germany followed by Wellcozyme Recombinant, anti HIV 1 Murex Biotech Ltd, Dart-ford, UK) in an alternative confirmatory strategy. [16] Samples with discrepant results were retested using Western blot (Genetic System, Redmond, WA), which was interpreted according to WHO criteria. [17] For the detection of *Treponema pallidum *antibodies, sera were first screened by Venereal Disease Research Laboratory (VDRL) test (Murex, Dartford, Kent, UK) and reactive sera were confirmed by *Treponema pallidum *Particle Agglutination (TPPA) assay (Fujirebio Diagnostics, Inc. Malvern, PA U.S.A.).

### Ethical issues

The study protocol was approved by the ethical review boards at Muhimbili University of Health and Allied Sciences and National Institute of Medical Research in Tanzania and Karolinska Institutet Syd, Stockholm, Sweden. All youth who were found to have an RTI were treated free-of-charge following the Ministry of Health and Social Welfare guidelines, while those who were found to be HIV infected were managed based on the prevailing guidelines in absence of antiretrovirals (ARVs) in Tanzania at that time. ARVs started being provided by the Ministry of Health and Social Welfare in accredited public and some private health care institutions in Tanzania during the second half of 2004.

### Statistical methods

Data were analysed using SPSS 12.0. Bivariate analyses were performed by cross tabulations. Proportions were compared using Chi-squared test. Comparison of means was done using the unpaired t test. The 25^th ^and 75^th ^percentiles of the median age differences between age at first sex and age of first contact were used as cut-off points for younger and older partners respectively. We examined sexual behavioural characteristics by the two categories of younger and older partners. Analysis was performed for males and females separately. A two tailed p-value < 0.05 was considered statistically significant.

## Results

Between April 2002 and June 2004, a total of 304 youth from the youth friendly STI clinic were recruited into the study out of whom 157(51.6%) were females. The reasons for visiting the clinic were; sickness in 193/304 (63.5%), HIV/STI voluntary testing and counselling in 55/304 (18.1%), being worried of their health in 46/304 (15.1%) while 2.0% were advised to come to the clinic by their contacts.

The social demographic characteristics of the study population are shown in Table 1. The majority of males were single and significantly older compared to females. Among the 44 married youth who attended the clinic all seven males and 25 of the 37 (67.6%) females visited the clinic due to ill health. Three males (2.0%) and ten females (6.4%) who came to the clinic reported that they had never engaged in sexual intercourse.

**Table 1:** Social demographic characteristics of youth attending a sexually transmitted infections clinic

**Characteristics**	**Males (n = 147)**	**Females (n = 157)**	**P value**
Mean (median, range) age	21.5(22.0, 11–24)	20.2(20.0, 13–24)	<0.01
Mean (median, range) age at menarche/spermarche	15.3(15.0, 9–16)	14.6(15.0,11–20)	0.01
Mean (median, range) age at coitarche	16.4(17.0, 9–22)	16.2(17.0, 13–24)	0.59
Mean (median, range) age of the first sexual partner	16.8(16.0, 7–32)	23.0(22.5, 14–40)	<0.01
Mean (median, range) no. of sex partners at present	0.9 (1.0,0–6)	0.9(1.0 0–4)	0.99
Mean (median, range) no. of sex partners last six months	1.7(1.0, 0–15)	1.2(1.0, 0–5)	<0.01
Mean (median, range) no. of lifetime sex partners	6.4(5.0 0–30)	2.8(2.0, 0–15)	<0.01
Single (%)	140 (95.2)	120(76.4)	<0.01
Petty business man (%)	76 (51.7)	26 (16.6)	<0.01
Students (%)	36 (24.5)	50 (31.8)	<0.01
Completed primary education (%)	92 (62.6)	82(52.2)	0.06
Completed secondary education (%)	44(29.9)	63(40.1)	0.06

The mean age at first sexual intercourse was 16.4 years in males compared to 16.2 years in females. The mean age of the first opposite sexual partner was significantly higher for female compared to male youth (23.0 vs16.8 years) (p < 0.01). The mean age difference between the age at sex initiation and the age of the first sexual partner was 0.4 years for male youth compared to 5.8 years for females (p < 0.01).

Seventy four (50.3%) males compared to 67 (42.7%) females had ever used condoms. For contraception, only 8.3%, 6.4%, 5.7%, and 1.3% of the sexually active female youth used depot provera, contraceptive pills, monthly calendar or intra-uterine devices respectively. Sixteen female youth had had 59 pregnancies of which 16 (27.1%) were unplanned. A total of 25 abortions were reported of which 10 were spontaneous while 15 (60%) were induced all of these were from unplanned pregnancies. In addition, 70 (44.6%) female youth reported to have had undergone procedures for menstrual regulation indicating that they had engaged in unprotected sexual intercourse which could have exposed them to unplanned pregnancies.

The first sexual intercourse was involuntary in 9/136 (6.6%) of male and 17/140 (12.1%) of female youth. Among the males, 7/9 (77.8%) of the involuntary sexual experiences were with an older perpetrator compared to 16/17 (94.1%) in the females. Among the 25 youth with involuntary first sexual intercourse; 21(80.8%) reported that the person was known to them.

There were no significant differences in the number of lifetime sexual partners, HIV prevalence, ever used condom or involved in involuntary first sexual intercourse among those who had younger sexual partners as compared to those with older sexual partners in both males and females. However, male youth with older partners were significantly more likely to have taken illicit drugs and alcohol compared to females (data not shown).

Sixty six (42.0%) female youth had received gifts/money for sexual favours compared to 14 (9.5%) males. In both males and females, those who received gifts/money had significantly higher mean number of lifetime sexual partners compared to those who did not. The prevalence of HIV infection was similar (12.1 vs 12.6%) among the two groups of female youth.

### Other sexual practices

Among the youth studied, 20/296 (6.7%) had ever practiced penile anal sex while 50/296 (16.9%) had ever practiced penile oral sex, and 17/291 (5.8%) had ever practiced oral-vaginal sex.

### HIV infection

HIV results were available from 120/147 (81.6%) of male youth and 124/157 (79.0%) of the female youth. There was no significant difference in the mean age and sex distribution among those youth with HIV results compared to those in which the test results were not available. The prevalence of HIV infection was 7.5% (9/120) in males compared to 15.3% (19/124) in the females (p = 0.04).

The prevalence of other STIs was; gonorrhoea 1/147 (0.68%), genital warts 7/214(3.3%), syphilis 2/147(1.4%), candidiasis 3/147(2.0%), pelvic inflammatory disease (PID) 8/140 (5.7%), genital ulcer disease (GUD) 19/139 (13.7%) and vaginal discharge syndrome (VDS) 32/139 (23.0%). Because of low numbers of the various STIs in the study group no attempts were made to relate them with HIV infection or sociodemographic characteristics.

## Discussion

It is well established that adolescents and younger youth engage in sexual practices which predispose them to STIs and unwanted pregnancies. These sexual practices have medical, social and psychological consequences. Our results indicate that more than 92% of studied youth had engaged in sexual activity before the age of 25 years, in agreement with other studies. [1,2,4]

On the other sexual practices, about 7% and 17% of the male and female youth were practising penile anal sex respectively. This again indicates the diversity of sexual practices among the youth some of which can increase the risk of acquiring STIs including HIV infection.

The age of the first sexual contact was similar in both male and female youth; however, female youth were more likely to have their sexual initiation with significantly older sexual partners compared to male youth. Among the reasons which have been given as to why female youth voluntarily selected older partner include seeking financial support, [1,2,4] or desire for intimacy and emotional security. [18-20] However, studies have also indicated that despite the support which they will get from the older sexual partner, females preferred monogamous rather than multiple partners. [20] Our results indicate that female youth were more than four times more likely to receive gifts/money for sexual favours than males. If the motive is both financial and social support then it is obvious that females are not likely to select same age sexual partners but rather older partners with or without considering the risk associated with such sexual relationships. Studies have shown that when female youth have relationships with older partners they are more likely to engage in risky practices such as sex without condoms due to increased power imbalance leading to male-controlled sexual decisions. [19]

It has also been shown that youth with older sexual partners are at increased risk of acquiring STIs. [12] We could however not demonstrate significant differences between those who reported older as compared to younger first sexual partners. The only significant difference was seen in male youth who had older female sexual partners who were more likely to be taking illicit drugs and alcohol.

This study also reflected low condom use among the studied youth; about 50% of males and 43% of females had ever used condoms. These figures are lower in males but similar in females when compared to rates reported earlier in a study conducted in the same clinic in 1997/1998 (68.8% and 37.1% for males and female youth respectively). [21] The low condom use among these youth is consistent with a recent report from Ethiopia indicating that 56.7% of secondary school students ever used condoms. [22]

On the other hand, the low utilization of contraceptive methods is a major concern and is reflected in the high number of unplanned pregnancies, most of which ended up in induced abortions (which are illegal in Tanzania). In addition, a high number of youth reported 'menstrual regulation', which could hide even more likelihood of abortions. In a study among adolescent girls with illegally induced abortions in Dar es Salaam, it was clearly shown that most adolescent girls were not aware of the national policy that gave them the right to seek family planning services but in practice these services were not being provided probably due to the nature of the environment where these services were being offered. [23] All of these put together, clearly identify missed opportunities and emphasize the need to have youth friendly reproductive health services which will include contraception. It is notable that the majority of the first sexual activities in the current study were voluntary, however when the first sexual act was involuntary, female youth were twice as likely to be the victims as males (12% vs 7.6%).

In this study, male youth were found to have a significantly higher number of sexual partners in the last six months or total number of lifetime sexual partners compared to female youth. However, neither the age at which the youth started sexual activity nor the age of the first sexual partner was associated with a higher number of sexual partners or higher prevalence of HIV infection. Among the males, alcohol and illicit drugs were associated with increased risk of being HIV infected.

Female youth reported a higher number of older male sexual partners. However, it was not apparent who the sexual partners of the young men were. There might be a core group of young females with many partners who do not access services at the STI clinic like the IDC. Reasons which have been suggested to explain the higher number of sexual partners that males report are; their desire for sexual gratification, to gain social status or over reporting in order to be respected as a man among their male peers to whom they look for guidance and support. [20] One approach to help these male youth will be to explore ways to facilitate social support and suggestions as to how to reduce the number of sexual partners.

There have been two other published reports from the same clinic from studies done in 1997/1998 and 1998/1999. [21,24] When the results from the current study are compared with the previous ones, there are indications of the same magnitude of at risk practices, but also an indication of the evolving HIV epidemic; the HIV prevalence in female youth was 24%, 21% and 15% in 1997/1998, 1998/1999 and 2002/2004 respectively while for male youth it was 5.9%, 12.0 and 7.5% respectively. [21,24] As the clinic has gradually become better known, an increasing number of youth are attending for advice rather than with STI symptoms. This is reflected in the relatively low number of detected STIs in the current study, however the prevalence of HIV and unwanted pregnancies continue to remain high.

## Limitations

Our findings are limited by including only youth attending the STI clinic, the cross sectional nature of the study, and reliance on reported sexual practices which are difficult to verify. The sample of studied youth may be biased in that it is a subpopulation of young people who are more sexually active and therefore more prone to seek advice on STI and HIV.

## Conclusion

We conclude that male and female youth are actively engaging in risky sexual practices at a relatively young age, female youth are having their first sexual intercourse with older male partners, and male youth have exceptionally high number of sexual partners. These results call for further studies but also strategies that will help youth on the consequences of these risky sexual practices, and also help them cope with these challenges including use of condoms, delay sexual initiation, and avoiding older sexual partners.

## Competing interests

The authors declare that they have no competing interests.

## Authors' contributions

WU participated in conduct of the study, performed data analysis, drafted and revised the manuscript. CM performed data analysis and revised the manuscript. GC participated in the concept design, conduct of the study and revision of the manuscript. FM conceived the study, supervised the conduct of the study and revised the manuscript. ES conceived the study, supervised the study and revised the manuscript. All authors read and approved the final manuscript.

## Pre-publication history

The pre-publication history for this paper can be accessed here:


